# Association between oxidative balance score and serum uric acid and hyperuricemia: a population-based study from the NHANES (2011–2018)

**DOI:** 10.3389/fendo.2024.1414075

**Published:** 2024-06-20

**Authors:** Yuhao Yang, Zengxiang Wu, Zhenmei An, Shuangqing Li

**Affiliations:** ^1^ General Practice Ward/International Medical Center Ward, General Practice Medical Center, West China Hospital, Sichuan University, Chengdu, China; ^2^ Department of Endocrinology and Metabolism, West China Hospital, Sichuan University, Chengdu, China

**Keywords:** oxidative balance score (OBS), Serum uric acid (sUA), hyperuricemia, national health and nutrition examination survey (NHANES), oxidative stress

## Abstract

**Background:**

Oxidative Balance Score (OBS) is a novel indicator of the overall antioxidant/oxidant balance, providing a comprehensive reflection of the body’s overall oxidative stress status, with higher OBS suggesting more substantial antioxidant exposures. We aimed to investigate the possible relationship between OBS with serum uric acid (SUA) and hyperuricemia.

**Methods:**

Data utilized in this study were sourced from the 2011–2018 National Health and Nutrition Examination Survey (NHANES). Participants under 18 years old, those with ≤16 complete data out of 20 OBS components, incomplete serum uric acid data, and missing covariates were excluded from the analysis. OBS was computed by evaluating 16 nutrients and 4 lifestyle factors, encompassing 5 pro-oxidants and 15 antioxidants, guided by *a priori* knowledge of their relationship with oxidative stress.

**Results:**

A total of 1,5096 individuals were included in our analysis with 49.7% being male, and an average age of 49.05 ± 17.56 years. The mean OBS was 19.76 ± 7.17. Hyperuricemia was present in 19.28% of participants. Due to the right-skewed distribution of the OBS, a natural log transformation was applied to address this issue, and Quartiles of lnOBS 1, 2, 3, and 4 were 1.10–2.56 (N=3526), 2.64–2.94 (N=3748), 3.00–3.22 (N=4026), and 3.26–3.61 (N=3796), respectively. Multivariable logistic regression showed that higher lnOBS quantiles were correlated with lower serum uric acid levels. Compared with the lowest lnOBS quantile, participants in the highest lnOBS quantile had a significant serum uric acid decrease of 16.94 μmol/L for each unit increase in lnOBS (β=-16.94, 95% CI: -20.44, -13.45). Similar negative associations were observed in the second-highest (β=-8.07, 95% CI: -11.45, -4.69) and third-highest (β=-11.69, 95% CI: -15.05, -8.34) lnOBS quantiles. The adjusted odds ratios (ORs) for hyperuricemia in Quartiles 1, 2, 3, and 4 were 1.00, 0.84 (95% CI: 0.75, 0.95), 0.78 (95% CI: 0.69, 0.88), and 0.62 (95% CI: 0.55, 0.71), respectively. Compared to Quartile 1, participants in Quartile 4 had a 38% lower prevalence of hyperuricemia. Subgroup analysis and interaction test showed that there was a significant dependence of sex between OBS and serum uric acid (p for interaction <0.05), but not hyperuricemia (p for interaction >0.05). Subgroup analysis stratified by age, BMI, hypertension, diabetes, and hyperlipidemia showed there is no significant dependence on these negative correlations (all p for interaction >0.05).

**Conclusions:**

The serum uric acid levels and prevalence of hyperuricemia in US adults exhibited a negative association with OBS. By exploring this connection, our research aims to gain a better understanding of how oxidative balance affects the prevalence of hyperuricemia. This could provide valuable insights for developing preventive strategies and interventions for hyperuricemia. Additional large-scale prospective studies are required to explore the role of OBS in hyperuricemia further.

## Introduction

Uric acid, the final oxidation product of purine metabolism, is primarily excreted by the kidneys. Elevated serum uric acid (SUA) levels can lead to hyperuricemia due to uric acid supersaturation (1). Hyperuricemia is commonly defined as serum uric acid concentrations exceeding 7.0 mg/dL in men or 6.0 mg/dL in women (2). While uric acid serves as a vital antioxidant in plasma, it also contributes to blood pressure regulation and resilience against oxidative stress (3). However, intracellular uric acid has been linked to elevated inflammation and heightened oxidative stress (4). Extensive research has identified high serum uric acid levels as an independent risk factor for various chronic kidney and joint disorders (5), including gout (6), chronic arthritis (7), joint deformity, and uric acid kidney stones (8), as well as chronic metabolic diseases such as hypertension (9), diabetes (10), and metabolic syndrome, along with cardiovascular disease (11).

Oxidative stress is a condition where the cell’s antioxidant scavenging system is overwhelmed by the overproduction of ROS, leading to an oxygen paradox. This paradox occurs when free radicals, necessary for cellular processes, are produced in excessive amounts and begin to interfere with essential metabolic processes (12). Current evidence suggests a correlation between hyperuricemia and elevated levels of oxidative stress, as evidenced by increased serum pro-oxidant-antioxidant balance values (13). Pro-oxidants are implicated in inducing oxidative stress through the generation of reactive oxygen species (ROS) or by impeding the defensive capacity of the antioxidant system (14). A multitude of studies have investigated the relationship between hyperuricemia and oxidative stress, establishing that elevated blood uric acid levels promote oxidative stress within the body (15), thereby triggering inflammation (16).

Despite this, there is a notable gap in the research exploring the fluctuations in individual antioxidant/oxidant balance status and their impact on the development of hyperuricemia. The OBS is an effective tool that allows us to evaluate an individual’s antioxidant status by ranking the antioxidant and pro-oxidant components of their diet and lifestyle factors. Numerous epidemiological studies have investigated the association between OBS and conditions such as diabetes, non-alcoholic fatty liver disease (17), periodontitis (18), lung health (19), and vascular endothelial function (20).

In this study, we aim to use data from the National Health and Nutrition Examination Survey (NHANES) to perform a cross-sectional analysis among adults in the United States. Our goal is to assess the relationship between OBS and hyperuricemia. By exploring this connection, our research aims to gain a better understanding of how oxidative balance affects serum uric acid levels and the prevalence of hyperuricemia. This could provide valuable insights for developing preventive strategies and interventions.

## Method

### Research question and hypothesis

The primary research question of this study is to investigate the association between OBS and hyperuricemia among adults in the United States. We hypothesize that higher OBS is associated with lower serum uric acid levels and a reduced risk of hyperuricemia.

### Data source

The NHANES has a complex multistage probabilistic sampling design. It annually examines a nationally representative sample of 5000 individuals at 15 different sites selected from a sampling frame of all counties in the United States (21). It is conducted by the National Center for Health Statistics (NCHS) and is a periodic survey approved by the NCHS Ethics Review Board. All participants provide informed consent before participation. NHANES data collection takes place throughout the year, including weekdays and weekends. It involves a household interview, a visit to a mobile examination center (MEC), and follow-up activities after the MEC visit. In the household screening interview, eligible household members are identified using a computer-assisted personal interview tool. Potential participants receive a comprehensive list of topics and categories, such as health examination, blood- and urine-based tests, and dietary intake, that will be assessed. After obtaining informed consent, a detailed in-person interview is conducted in the homes of consenting individuals. This interview covers demographic, socioeconomic, dietary (including supplement use), and health-related questions (22). The data are released in 2-year cycles. four consecutive NHANES 2-year cycles (2011–2012, 2013–2014, 2015–2016, and 2017–2018) are collected. All NHANES data are publicly available at https://www.cdc.gov/nchs/nhanes/.

### Study participants

**Figure 1:**
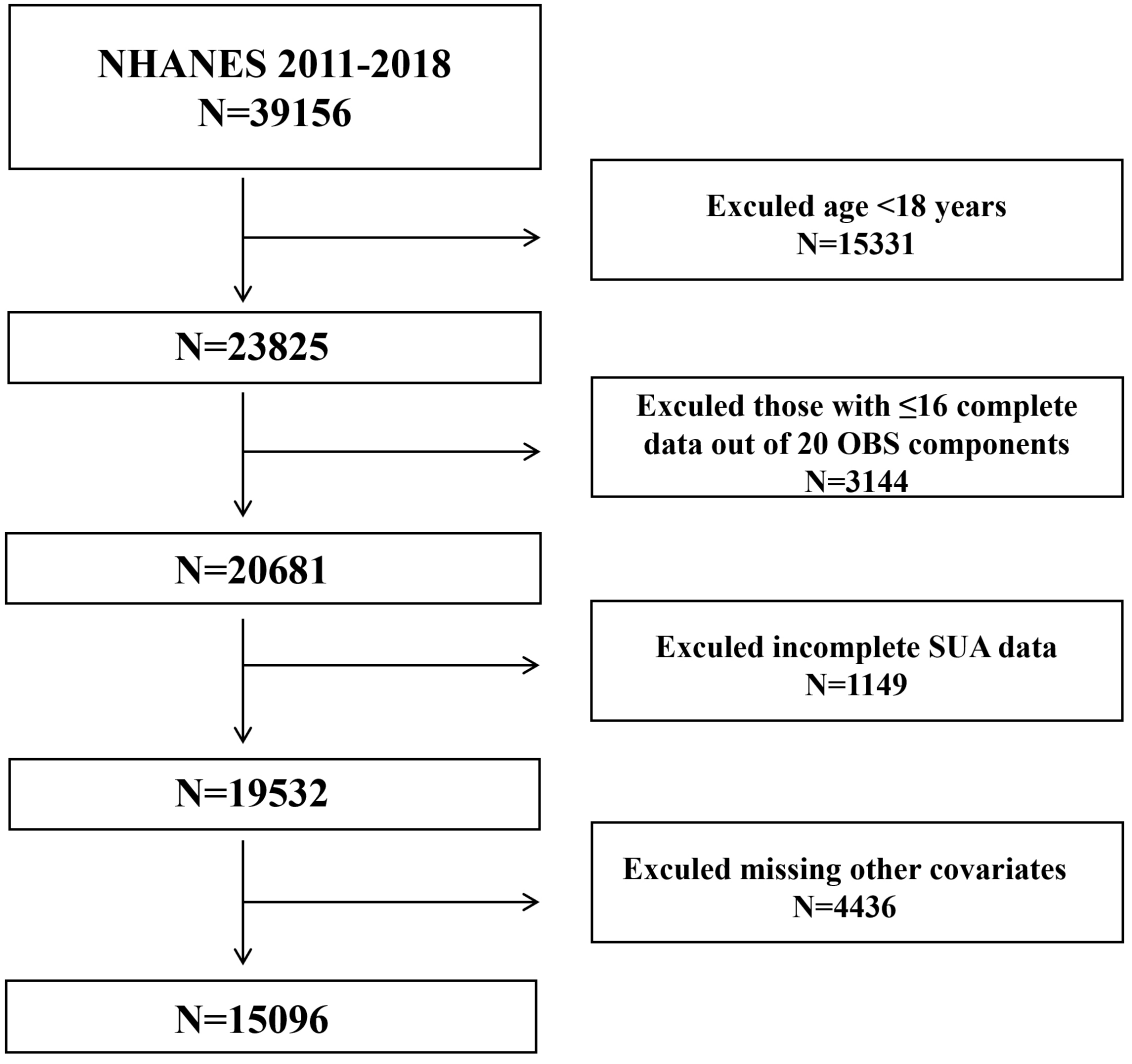
Flow diagram of clinical research from NHANES 2011–2018.

From the NHANES 2011−2018 dataset, a total of 39,156 individuals were identified to investigate the relationship between OBS and hyperuricemia. Exclusion criteria comprised individuals below 18 years of age (n=15,331), those with ≤16 complete data out of 20 OBS components (n=3,144), incomplete SUA data (n=1,149), and missing other covariates (n=4,436). Ultimately, 15,096 eligible participants were included in the final analysis (**Figure 1**).

### Outcome variables

Serum samples were obtained from participants and stored at –30°C until transported to the National Center for Health Statistics (NCHS) at the Centers for Disease Control and Prevention (CDC) for serum uric acid (SUA) testing. Hyperuricemia was defined as an SUA level ≥416 μmol/L (7 mg/dL) in men and ≥357 μmol/L (6 mg/dL) in women (23).

### Exposure assessment

The OBS was computed by assessing 16 nutrients and 4 lifestyle factors, comprising 5 pro-oxidants and 15 antioxidants, based on *a priori* knowledge of their association with oxidative stress. OBS components were categorized into four groups: (1) dietary antioxidants (fiber, β-carotene, riboflavin, niacin, vitamin B6, total folate, vitamin B12, vitamin C, vitamin E, calcium, magnesium, zinc, copper, and selenium), (2) dietary pro-oxidants (total fat and iron), (3) lifestyle antioxidants (physical activity), and (4) lifestyle pro-oxidants (alcohol, smoking, and body mass index [BMI]). Alcohol consumption was stratified into three groups: heavy drinkers (≥15 g/d for women and ≥30 g/d for men), non-heavy drinkers (0–15 g/d for women and 0–30 g/d for men), and non-drinkers, with corresponding scores of 0, 1, and 2 points, respectively. Subsequently, other components were stratified by sex and divided into tertiles. Antioxidants were assigned points on a scale from 0 to 2 for tertile groups 1 to 3, respectively, with higher points indicating increased antioxidant levels. Conversely, pro-oxidants were assigned points inversely, with 0 points for the highest tertile and 2 points for the lowest tertile, reflecting higher pro-oxidant levels (24). Refer to **Table 1** for the specific calculation method of OBS. The overall OBS was calculated by summing the points assigned for each component, ranging from 3 to 37, with higher scores indicating greater antioxidant exposure.

**Table 1:** Ingredients that make up the oxidative balance score.

OBS components	Property	Male			Female		
		0	1	2	0	1	2
Dietary OBS components							
Dietary fiber (g/d)	A	≤13.20	13.20–20.80	>20.80	≤11.30	11.30–17.30	>17.30
Carotene (RE/d)	A	≤53.58	53.58–176.81	>176.81	≤57.60	57.60–198.53	>198.53
Riboflavin (mg/d)	A	≤1.80	1.80–2.64	>2.64	≤1.40	1.40–2.02	>2.02
Niacin (mg/d)	A	≤22.93	22.93–32.73	>32.73	≤16.29	16.29–23.49	>23.49
Vitamin B6 (mg/d)	A	≤1.74	1.74–2.59	>2.59	≤1.29	1.29–1.89	>1.89
Total folate (mcg/d)	A	≤328.33	328.33–495	>495.00	≤257.39	257.39–385.50	>385.50
Vitamin B12 (mcg/d)	A	≤3.66	3.66–4.40	>4.40	≤2.56	2.56–4.50	>4.50
Vitamin C (mg/d)	A	≤43.23	43.23–104.13	>104.13	≤41.45	41.45–90.35	>90.35
Vitamin E (ATE) (mg/d)	A	≤6.02	6.02–9.57	>9.57	≤5.03	5.03–7.98	>7.98
Calcium (mg/d)	A	≤751.00	751.00–1130.50	>1130.50	≤610.67	610.67–921.50	>921.50
Magnesium (mg/d)	A	≤262.33	262.33–366.50	>366.50	≤211.00	211.00–293.00	>293.00
Zinc (mg/d)	A	≤10.00	10.00–14.65	>14.65	≤7.24	7.24–10.54	>10.54
Copper (mg/d)	A	≤1.08	1.08–1.53	>1.53	≤0.88	0.88–1.25	>1.25
Selenium (mcg/d)	A	≤102.60	102.6–145.48	>145.48	≤74.77	74.77–105.73	>105.73
Total fat (g/d)	P	≤70.92	70.92–104.62	>104.62	≤53.03	53.03–77.44	>77.44
Iron (mg/d)	P	≤12.72	12.72–18.74	>18.74	≤9.79	9.79–14.12	>14.12
Lifestyle OBS components							
Physical activity	A	Low	Moderate	High	Low	Moderate	High
Alcohol (g/d)	P	≥30	0–30	None	≥15	0–15	None
Obesity	P	Obesity	Overweight	Normal	Obesity	Overweight	Normal
Smoking status	P	Current smoker	Former smoker	Never smoker	Current smoker	Smoking status	Never smoker

OBS, oxidative balance score; A, antioxidant; P, prooxidant; RE, retinol equivalent; ATE, alpha-tocopherol equivalent; MET, metabolic equivalent.

### Covariates

Our study incorporated various covariates potentially influencing the outcome, including gender, age, race, education level, poverty-to-income ratio (PIR), body mass index (BMI), drinking status, smoking status, hypertension, diabetes, and hyperlipidemia. Race/ethnicity categories comprised Non-Hispanic White, Non-Hispanic Black, Mexican American, Other Hispanic, or Other Race. Body mass index (BMI) was calculated as weight in kilograms divided by height in meters squared and categorized as underweight (<18.5 kg/m²), normal weight (18.5–24.9 kg/m²), overweight (25.0–29.9 kg/m²), and obese (≥30.0 kg/m²). Education level was classified as less than 9th grade, 9–11th grade, high school graduate, college degree, and college graduate or above. Smoking status was categorized as never smoking (defined as smoking < 100 cigarettes in life) or current smoking (defined as smoking ≥ 100 cigarettes in life) while drinking status was divided into non-drinking (defined as drinking < 12 times in the last year) or drinking (defined as drinking ≥ 12 times in the last year). Information on the prevalence of hypertension, diabetes, and hyperlipidemia among participants was obtained through self-reported questionnaires.

### Statistical analysis

All statistical analyses followed the guidelines outlined by the Centers for Disease Control and Prevention (CDC). Continuous variables were reported as means with standard error (SE), while categorical variables were presented as proportions. Differences among participants stratified by OBS quantiles were evaluated using either a weighted Student’s t-test (for continuous variables) or a weighted chi-square test (for categorical variables). Multivariable logistic regression, adjusted for various covariates, was employed to investigate the association between continuous OBS or quartile OBS and SUA levels, as well as hyperuricemia. Model 1 represented the unadjusted model, Model 2 was adjusted for age, sex, and race/ethnicity, and Model 3 adjusted for all covariates. Beta (β) coefficients with 95% confidence intervals (CIs) were used to evaluate the association between OBS and SUA levels. In comparison, odds ratios (ORs) with 95% confidence intervals (CIs) were used to assess the association between OBS and hyperuricemia. Subgroup analyses were conducted to explore the association between OBS and SUA levels, and hyperuricemia across gender, age, BMI, hypertension, diabetes, and hyperlipidemia subgroups. Due to the right-skewed distribution of OBS, natural log transformation was applied, and OBS values were categorized into four subgroups based on quartiles for inclusion in the models as continuous and categorical variables.

The statistical study was carried out using the statistical computing and graphics software R (version 4.1.3) and EmpowerStats (version 2.0).

## Results

### Baseline characteristics of participants

**Figure 2:**
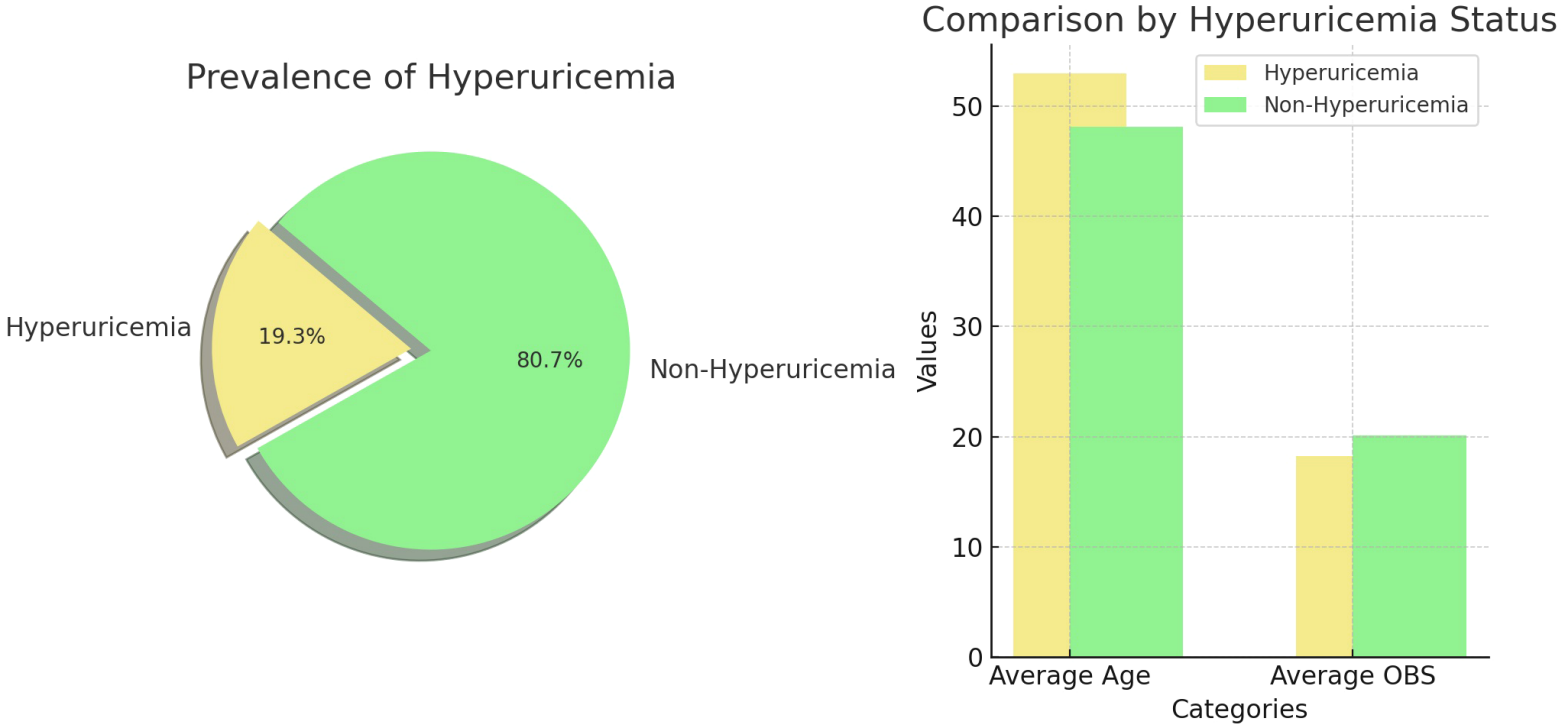
Hyperuricemia Prevalence and Demographic Analysis.

A total of 15,096 participants were enrolled in the study, with 49.7% being male, and an average age of 49.05 ± 17.56 years. The mean OBS concentration was 19.96 ± 7.17. When the population was divided into hyperuricemia and non-hyperuricemia groups, we found that hyperuricemia was present in 19.28% (N=2911) of participants. The average age of individuals with hyperuricemia was 52.96 ± 17.72 years, with an average OBS of 18.22 ± 7.03. In contrast, the average age of individuals without hyperuricemia was 48.12 ± 17.40 years, with an average OBS of 20.12 ± 7.15 (**Figure 2**). The clinical characteristics of participants, stratified by hyperuricemia status, are detailed in **Table 2**. We can use the education level as an example. Among participants with hyperuricemia, those with an education level of 9–11th grade accounted for 10.96% (N=319) of the total education levels (N=2911). In contrast, among participants without hyperuricemia, those with an education level of 9–11th grade accounted for 12.00% (N=1462) of the total education levels (N=12185). Significant associations were observed between hyperuricemia and age, sex, race, education level, BMI, hypertension, hyperlipidemia, diabetes, smoking status, and OBS (p < 0.05). Participants with hyperuricemia tended to be older, male, obese, have lower OBS, and have a higher proportion of hypertension, hyperlipidemia, diabetes, and smoking status compared to those without hyperuricemia.

**Table 2:** Baseline characteristics of participants according to hyperuricemia.

Characteristic	non-hyperuricemia	hyperuricemia	P value
	N=12185	N=2911	
Age(years)	48.12 ± 17.40	52.96 ± 17.72	<0.001
Sex,%			<0.001
Male	5818 (47.75%)	1685 (57.88%)	
Female	6367 (52.25%)	1226 (42.12%)	
Race,%			<0.001
Mexican American	1713 (14.06%)	282 (9.69%)	
Other Hispanic	1287 (10.56%)	230 (7.90%)	
Non-Hispanic White	4983 (40.89%)	1237 (42.49%)	
Non-Hispanic Black	2475 (20.31%)	749 (25.73%)	
Other Race	1727 (14.17%)	413 (14.19%)	
BMI,%(kg/m^2^)			<0.001
<18.5	208 (1.71%)	11 (0.38%)	
18.5–24.9	3652 (29.97%)	370 (12.71%)	
25.0–29.9	4024 (33.02%)	847 (29.10%)	
≥30	4301 (35.30%)	1683 (57.82%)	
Education Level,%			<0.001
Less than 9th grade	937 (7.69%)	209 (7.18%)	
9–11th grade	1462 (12.00%)	319 (10.96%)	
High school graduate	2718 (22.31%)	715 (24.56%)	
College degree	3850 (31.60%)	994 (34.15%)	
College graduate or above	3218 (26.41%)	674 (23.15%)	
PIR	2.53 ± 1.63	2.52 ± 1.62	0.929
Hypertension,%			<0.001
Yes	3873 (31.78%)	1587 (54.52%)	
No	8312 (68.22%)	1324 (45.48%)	
Hyperlipidemia,%			<0.001
Yes	4021 (33.00%)	1252 (43.01%)	
No	8164 (67.00%)	1659 (56.99%)	
Diabetes,%			<0.001
Yes	1553 (12.75%)	580 (19.92%)	
No	10632 (87.25%)	2331 (80.08%)	
Smoking Status,%			<0.001
Yes	5298 (43.48%)	1384 (47.54%)	
No	6887 (56.52%)	1527 (52.46%)	
Drinking Status,%			0.940
Yes	8032 (65.92%)	1921 (65.99%)	
No	4153 (34.08%)	990 (34.01%)	
OBS	20.12 ± 7.15	18.22 ± 7.03	<0.001

**Figure 3:**
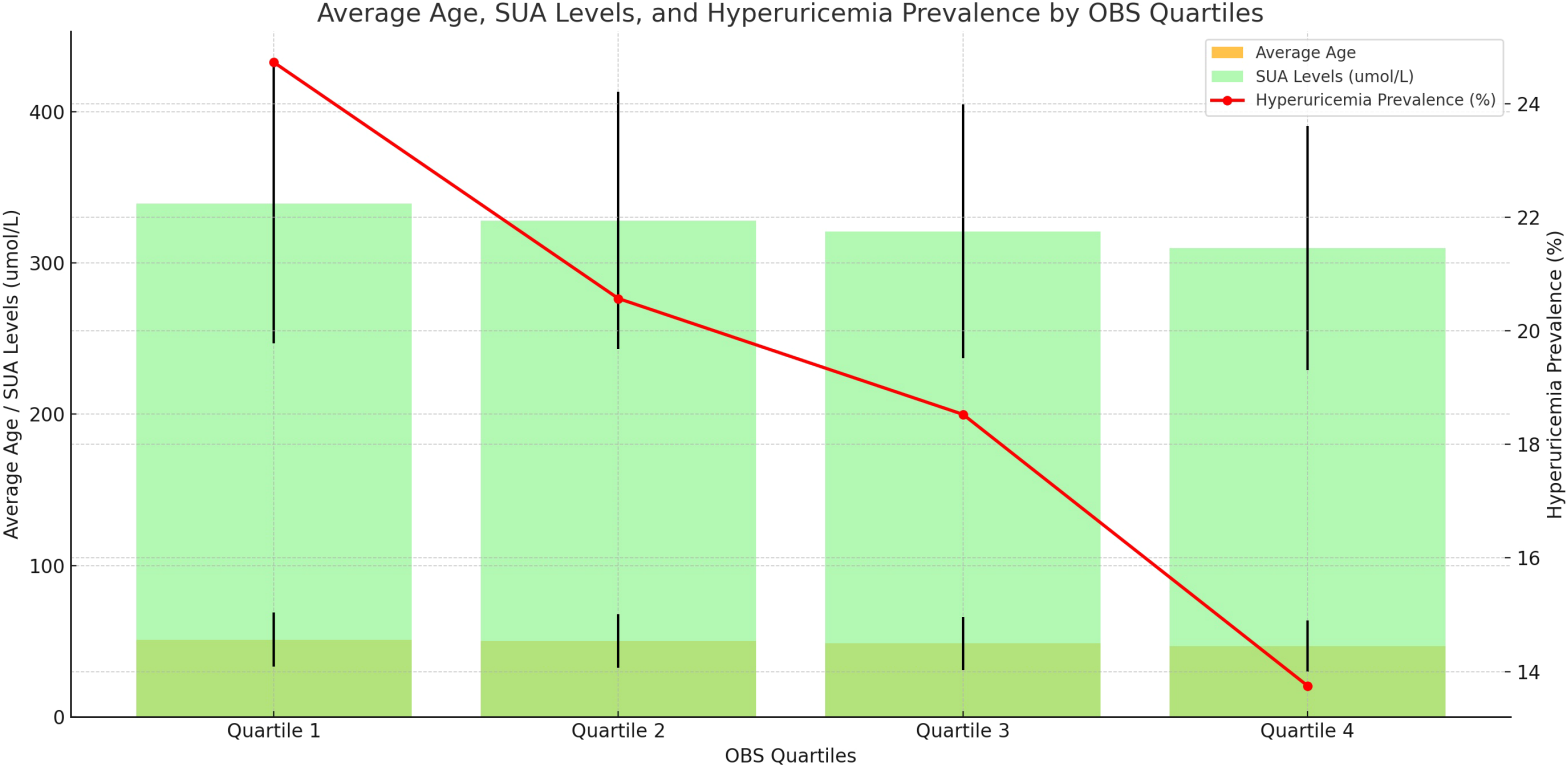
Distribution of Average Age, Serum Uric Acid Levels, and Hyperuricemia Prevalence Across OBS Quartiles.

**Table 3** displays the clinical characteristics of participants based on quartiles of OBS. The mean OBS was 19.96 ± 7.17, ranging from 3 to 37. Quartiles of OBS 1, 2, 3, and 4 were 3–13 (N=3526), 14–19 (N=3748), 20–25 (N=4026), and 26–37 (N=3796), respectively. The average age of individuals in Quartiles 1–4 was 50.98 ± 17.88, 50.02 ± 17.73, 48.57 ± 17.44, and 46.82 ± 16.95. The SUA levels of Quartiles 1–4 were 339.15 ± 92.37 umol/L, 327.89 ± 84.98 umol/L, 320.90 ± 83.99 umol/L, and 309.84 ± 80.71 umol/L, respectively. We observed that compared to Quartile 1 (24.73%), the prevalence of hyperuricemia was lower in Quartile 2 (20.57%), Quartile 3 (18.53%), and Quartile 4 (13.75%) (**Figure 3**). Significant differences were observed across OBS quartiles in age, race, BMI, SUA levels, PIR, education level, hypertension, hyperlipidemia, diabetes, hyperuricemia, drinking status, and smoking status (all p < 0.05). Participants in higher OBS quartiles tended to be younger, have higher income, normal weight, lower SUA levels, and a lower proportion of hypertension, hyperlipidemia, diabetes, hyperuricemia, and smoking status, with a higher proportion of drinking status compared to those in the lowest OBS quartile (p < 0.05).

**Table 3:** Baseline characteristics of participants according to the oxidative balance score’s quartile.

Characteristic	Quartiles of OBS				P-value
	Q1(N=3526)	Q2(N=3748)	Q3(N=4026)	Q4(N=3796)	
Age(years)	50.98 ± 17.88	50.02 ± 17.73	48.57 ± 17.44	46.82 ± 16.95	<0.001
SUA(umol/L)	339.15 ± 92.37	327.89 ± 84.98	320.90 ± 83.99	309.84 ± 80.71	<0.001
Gender,%					0.163
Male	1793 (50.85%)	1890 (50.43%)	1958 (48.63%)	1862 (49.05%)	
Female	1733 (49.15%)	1858 (49.57%)	2068 (51.37%)	1934 (50.95%)	
Race,%					<0.001
Mexican American	372 (10.55%)	486 (12.97%)	559 (13.88%)	578 (15.23%)	
Other Hispanic	317 (8.99%)	395 (10.54%)	426 (10.58%)	379 (9.98%)	
Non-Hispanic White	1362 (38.63%)	1533 (40.90%)	1688 (41.93%)	1637 (43.12%)	
Non-Hispanic Black	1092 (30.97%)	834 (22.25%)	743 (18.46%)	555 (14.62%)	
Other Race	383 (10.86%)	500 (13.34%)	610 (15.15%)	647 (17.04%)	
BMI,%(kg/m^2^)					<0.001
<18.5	56 (1.59%)	41 (1.09%)	50 (1.24%)	72 (1.90%)	
18.5–24.9	692 (19.63%)	906 (24.17%)	1076 (26.73%)	1348 (35.51%)	
25.0–29.9	1101 (31.23%)	1213 (32.36%)	1316 (32.69%)	1241 (32.69%)	
≥30	1677 (47.56%)	1588 (42.37%)	1584 (39.34%)	1135 (29.90%)	
Education Level,%					<0.001
Less than 9th grade	322 (9.13%)	325 (8.67%)	297 (7.38%)	202 (5.32%)	
9–11th grade	578 (16.39%)	453 (12.09%)	433 (10.76%)	317 (8.35%)	
High school graduate	959 (27.20%)	891 (23.77%)	908 (22.55%)	675 (17.78%)	
College degree	1144 (32.44%)	1240 (33.08%)	1292 (32.09%)	1168 (30.77%)	
College graduate or above	322 (9.13%)	325 (8.67%)	297 (7.38%)	202 (5.32%)	
PIR	2.10 ± 1.50	2.47 ± 1.60	2.62 ± 1.63	2.87 ± 1.68	<0.001
Hypertension Status,%					<0.001
Yes	1504 (42.65%)	1437 (38.34%)	1412 (35.07%)	1107 (29.16%)	
No	2022 (57.35%)	2311 (61.66%)	2614 (64.93%)	2689 (70.84%)	
High Cholesterol Status,%					<0.001
Yes	1269 (35.99%)	1374 (36.66%)	1414 (35.12%)	1216 (32.03%)	
No	2257 (64.01%)	2374 (63.34%)	2612 (64.88%)	2580 (67.97%)	
Diabetes Status,%					<0.001
Yes	641 (18.18%)	582 (15.53%)	534 (13.26%)	376 (9.91%)	
No	2885 (81.82%)	3166 (84.47%)	3492 (86.74%)	3420 (90.09%)	
Smoking Status,%					<0.001
Yes	1861 (52.78%)	1740 (46.42%)	1672 (41.53%)	1409 (37.12%)	
No	1665 (47.22%)	2008 (53.58%)	2354 (58.47%)	2387 (62.88%)	
Drinking Status,%					<0.001
Yes	2223 (63.05%)	2442 (65.15%)	2657 (66.00%)	2631 (69.31%)	
No	1303 (36.95%)	1306 (34.85%)	1369 (34.00%)	1165 (30.69%)	
Hyperuricemia					<0.001
Yes	872 (24.73%)	771 (20.57%)	746 (18.53%)	522 (13.75%)	
No	2654 (75.27%)	2977 (79.43%)	3280 (81.47%)	3274 (86.25%)	

### Association between lnOBS and SUA

A natural log transformation was applied to address the right-skewed distribution of the OBS and Quartiles of lnOBS 1, 2, 3, and 4 were 1.10–2.56 (N=3526), 2.64–2.94 (N=3748), 3.00–3.22 (N=4026), and 3.26–3.61 (N=3796), respectively. **Table 4** shows the results of the multivariate regression analysis. In the unadjusted model, lnOBS exhibited a negative association with SUA levels (β=25.33, 95% CI: -28.59, -22.06). Even after full adjustment for age, sex, body mass index (BMI), race/ethnicity, educational level, smoking, drinking, diabetes, hypertension, and hyperlipidemia, the negative association between lnOBS and SUA levels remained consistent (β=-14.47, 95% CI: -17.41, -11.53). This indicated that each unit increase in lnOBS was associated with a decrease of 14.47 μmol/L in SUA levels, suggesting that higher OBS was correlated with lower SUA levels. We further converted the lnOBS from a continuous variable to a categorical variable (quantiles) to conduct a comparison. Compared with the lowest lnOBS quantile, participants in the highest lnOBS quantile had a significant serum uric acid decrease of 16.94 μmol/L when each unit of lnOBS increased with statistical significance (β=-16.94, 95% CI: -20.44, -13.45). Similar negative associations were observed in the second-highest (β=-8.07, 95% CI: -11.45, -4.69) and third-highest (β=-11.69, 95% CI: -15.05, -8.34) lnOBS quantiles.

**Table 4:** Multiple logistic regression associations of lnOBS with SUA in adults.

lnOBS	Model 1 β (95% CI) P value	Model 2 β (95% CI) P value	Model 3 β (95% CI) P value
Continuous	-25.33 (-28.59, -22.06) <0.0001	-21.69 (-24.70, -18.68) <0.0001	-14.47 (-17.41, -11.53) <0.0001
Categories			
Q1	0	0	0
Q2	-11.27 (-15.20, -7.34) <0.0001	-9.80 (-13.37, -6.23) <0.0001	-8.07 (-11.45, -4.69) <0.0001
Q3	-18.26 (-22.12, -14.39) <0.0001	-14.61 (-18.13, -11.08) <0.0001	-11.69 (-15.05, -8.34) <0.0001
Q4	-29.31 (-33.23, -25.39) <0.0001	-24.86 (-28.46, -21.26) <0.0001	-16.94 (-20.44, -13.45) <0.0001

OBS, oxidative balance score; SUA, serum uric acid; Q, quartile; CI, Confidence Interval.

### Association between lnOBS and hyperuricemia

**Table 5** shows that a negative association was observed between lnOBS and hyperuricemia (OR 0.68, 95% CI 0.61, 0.75) in multiple logistic regression analysis adjusting for all covariates. This indicates that a one-unit increase in lnOBS is associated with a 32% lower prevalence of hyperuricemia. When lnOBS was categorized into quartiles, similar negative associations were observed compared to continuous lnOBS. The adjusted odds ratios (ORs) for Quartiles 1, 2, 3, and 4 in model 3 were 1.00, 0.84 (95% CI 0.75, 0.95), 0.78 (95% CI 0.69, 0.88), and 0.62 (95% CI 0.55, 0.71), respectively. Compared to Quartile 1, participants in Quartile 4 had a 38% lower prevalence of hyperuricemia.

**Table 5:** Multiple logistic regression associations of lnOBS with hyperuricemia in adults.

lnOBS	Model 1 OR (95% CI) P value	Model 2 OR (95% CI) P value	Model 3 OR (95% CI) P value
Continuous	0.55 (0.50, 0.60) <0.0001	0.59 (0.54, 0.65) <0.0001	0.68 (0.61, 0.75) <0.0001
Categories			
Q1	1.0(ref)	1.0(ref)	1.0(ref)
Q2	0.79 (0.71, 0.88) <0.0001	0.82 (0.73, 0.91) 0.0004	0.84 (0.75, 0.95) 0.0039
Q3	0.69 (0.62, 0.77) <0.0001	0.75 (0.67, 0.83) <0.0001	0.78 (0.69, 0.88) <0.0001
Q4	0.49 (0.43, 0.55) <0.0001	0.54 (0.47, 0.61) <0.0001	0.62 (0.55, 0.71) <0.0001

OBS, oxidative balance score; Q, quartile; CI, Confidence Interval; ref, reference category.

### Subgroup analysis for the association between lnOBS and SUA

**Figure 4:**
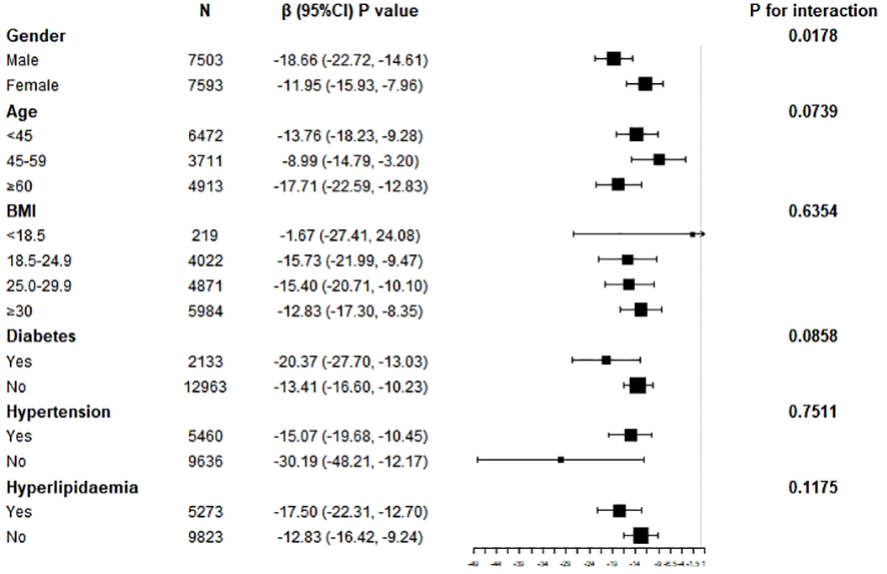
Subgroup analysis for the association between OBS and SUA in adults.

We conducted a subgroup analysis to assess the stability of the relationship between lnOBS and SUA levels across different population settings (**Figure 4**). Stratification by gender revealed a significant and independently negative association between lnOBS and SUA levels in both men and women (p for interaction < 0.05). However, for other stratifications including age, BMI, hypertension, diabetes, and hyperlipidemia status, the negative association between lnOBS and SUA levels remained unaffected and was not significantly influenced (p for interaction > 0.05).

### Subgroup analysis for the association between lnOBS and hyperuricemia

**Figure 5:**
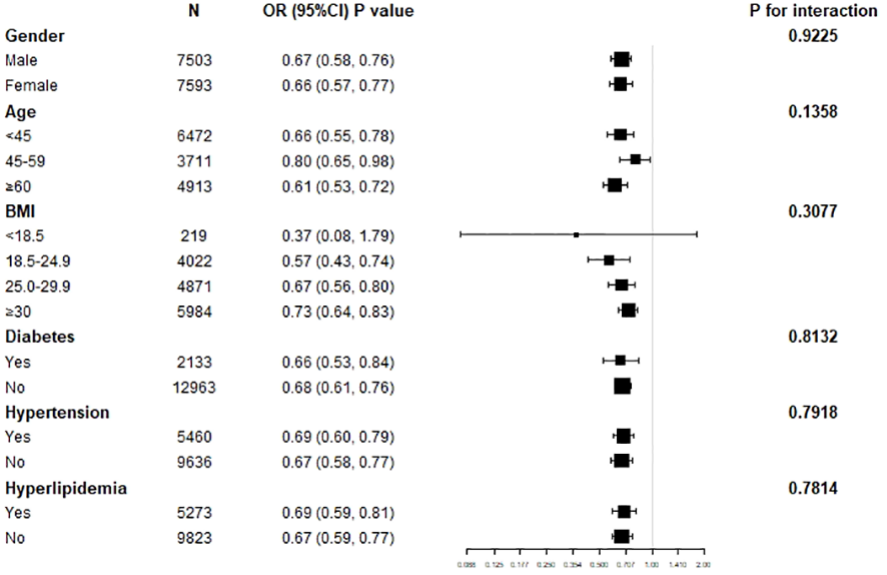
Subgroup analysis for the association between OBS and hyperuricemia in adults.

Further stratified analyses between lnOBS and hyperuricemia were conducted (**Figure 5**). These analyses indicated that gender, age, BMI, hypertension, diabetes, and hyperlipidemia status did not significantly influence the modification of the association between lnOBS and hyperuricemia (all P for interaction > 0.05).

## Discussion

In the cross-sectional study involving 15,096 participants, our research demonstrated a significant negative association between OBS and both SUA and hyperuricemia, indicating that increased OBS may contribute to lower SUA levels and decreased hyperuricemia prevalence. The association remained stable after covariate adjustment. Subgroup analysis stratified by sex, age, BMI, hypertension, diabetes, and hyperlipidemia showed that there is a significant sex dependence on lowering SUA levels but not hyperuricemia. No significant dependence was observed for age, BMI, hypertension, diabetes, or hyperlipidemia.

As far as we know, this is the first study to assess the relationship between OBS and both SUA and hyperuricemia based on NHANES (from 2011 to March 2018). Previous studies have explored the relationship between oxidative stress and SUA (25) or hyperuricemia (16). The dietary components of OBS include many ingredients, and many of them have been proven to have antioxidant effects in previous studies. Studies found increasing dietary fiber intake could increase probiotics in the gut which is closely related to the occurrence and development of hyperuricemia by participating in the synthesis of purine metabolizing enzymes and the release of inflammatory factors (26). Some clinical studies support the beneficial role of vitamin B, C, D, and E supplementation in reducing SUA levels amongst healthy adults and, consequently, hyperuricemia (25, 27–29). A cross-sectional study presented a negative correlation between dietary magnesium intake and hyperuricemia in both males and females among US adults after adjusting for major confounding factors (30). In addition, a basic study also suggested that Cu or Cu-containing compounds significantly inhibited xanthine dehydrogenase (XDH) activity and reduced uric acid production by XDH in mouse liver homogenates (31). A significant positive association between higher serum iron and the risk of hyperuricemia was demonstrated in an observational cohort study of different regions of the Chinese population (32). However, some recent studies suggest that antioxidants do not provide significant benefits to the body and may even pose certain health risks. For instance, a prospective study involving 345,000 people found no significant association between antioxidant use and all-cause, cancer, or non-cancer mortality (33). Additionally, other research indicates that antioxidant supplementation may interfere with the body’s defense mechanisms and possess pro-oxidant properties, potentially increasing the risk of cancer and cardiovascular diseases (34). Therefore, it is important to note that a single component of trace elements may not be adequate to fully elucidate its antioxidant effects on the body.

The impact of lifestyle on hyperuricemia has been extensively explored in numerous studies. A cross-sectional analysis involving 38,855 participants revealed that higher sitting time was independently associated with an increased prevalence of hyperuricemia, whereas engaging in vigorous physical activity was associated with a decreased prevalence of hyperuricemia (35). Additionally, a Mendelian randomization study conducted on Korean hyperuricemic individuals indicated a significant causal relationship between increased alcohol consumption and the incidence of hyperuricemia (36). Furthermore, two nationwide cross-sectional surveys of the non-institutionalized Korean population suggested that cotinine-verified smoking was significantly associated with serum uric acid levels in women, and the risk of hyperuricemia increased in a dose-response manner with higher smoking exposure (37, 38). Additionally, we observed a positive association between obesity and hyperuricemia, which appears to be mediated by insulin resistance (39). In this study, the OBS serves as an indicator of the overall antioxidant/oxidant balance, providing a more comprehensive reflection of the body’s overall oxidative stress status (40).

Oxidative stress denotes a pathological imbalance between pro-oxidative and anti-oxidative factors, favoring the generation of pro-oxidants, which often manifests as an overproduction of reactive oxygen species (ROS) (14). Chronic inflammation leads to a pro-oxidative state, which is closely linked to the development or progression of cardiovascular disease (CVD) (41), hypertension (42) and metabolic syndrome (43). In many disease states, the expression of oxidase-producing enzymes, the primary sources of ROS, is upregulated (44). In humans and great apes, uric acid represents the end product of purine metabolism, with xanthine oxidase (XOR) acting as the rate-limiting enzyme in purine catabolism (45). XOR catalyzes the final two steps of purine catabolism, converting hypoxanthine to xanthine and then to uric acid, a process accompanied by ROS production, thereby contributing to oxidative stress (46). This oxidative stress can lead to damage to proteins, lipids, DNA, and RNA, and participate in various cellular processes such as cell signaling, cardiovascular disease, inflammation, aging, and cancer (47). The intricate relationship between oxidative stress and serum uric acid concentration has been the focus of research for decades (16). Numerous observational cross-sectional studies have affirmed the association of individual pro-oxidative and oxidative factors with uric acid and hyperuricemia. However, determining the precise impact of individual oxidative stress-related components on serum uric acid poses a considerable challenge (18). Moreover, pro-oxidants and antioxidants may exhibit antagonistic or synergistic effects. Furthermore, antioxidants can promote oxidation at high doses, while exhibiting poor solubility, low permeability, and lacking stability and specificity in biofilms (48). Hence, we employed the OBS as a comprehensive evaluation index to assess individual oxidative balance. Our findings revealed a negative correlation between higher OBS and SUA levels, suggesting that elevated OBS is associated with a reduced prevalence of hyperuricemia.

This phenomenon can be explained through the following mechanisms: 1. Impact of Antioxidants on Uric Acid Production: Antioxidants such as vitamin C (49), vitamin E (50), and zinc (51) have been shown to reduce serum uric acid levels. Vitamin C, for example, can alter the activity of URAT1 in renal tubular epithelial cells, promoting uric acid excretion and thus lowering serum uric acid levels (52, 53). Antioxidants reduce uric acid production by inhibiting pathways that generate uric acid. 2. Relief of Oxidative Stress by Antioxidants: Oxidative stress, characterized by an imbalance between ROS production and antioxidant defense, is closely related to hyperuricemia (46). Behaviors such as smoking and alcohol consumption increase oxidative stress, and antioxidants can neutralize these oxidants, reducing oxidative damage to cells. Since uric acid has antioxidant properties, reduced oxidative stress would decrease the body’s need for uric acid as an antioxidant, thus lowering serum uric acid levels (36, 38). 3. Protection of Renal Function: Antioxidants protect renal function by preventing oxidative damage to glomeruli and renal tubules. The kidneys play a crucial role in uric acid excretion, and renal damage can directly affect uric acid clearance. By preserving renal function, antioxidants facilitate normal uric acid excretion, thereby reducing serum uric acid levels (54, 55). 4. Improvement of Metabolism: The OBS includes antioxidants that play a significant role in improving insulin resistance and metabolic syndrome. Insulin resistance is closely linked to hyperuricemia because it increases uric acid production and decreases its excretion. By improving insulin sensitivity, antioxidants help maintain normal uric acid metabolism and reduce the risk of hyperuricemia (56, 57). 5. Antioxidants and Gut Health Synergistic Uric Acid Reduction: The intake of antioxidants and gut health have a closely linked bidirectional relationship, which plays a crucial role in reducing uric acid levels and preventing hyperuricemia. Antioxidants reduce oxidative stress and inflammation-induced damage to intestinal epithelial cells, thereby decreasing intestinal permeability, promoting cell repair, and enhancing gut barrier function. Additionally, antioxidants inhibit the production and release of inflammatory mediators, protecting the gut microbiota and maintaining the balance and integrity of the gut flora (58–60). A healthy gut environment aids in the absorption and utilization of antioxidants, further enhancing their bioactivity. Certain gut microbes can metabolize antioxidants, converting them into more effective forms, thus boosting their antioxidant effects (61–63). This positive feedback loop not only helps lower uric acid levels in the body but also promotes uric acid excretion by regulating uric acid transporters in the gut, effectively preventing and controlling hyperuricemia.

Subgroup analysis and interaction tests were employed to observe gender differences in the effect of OBS on SUA, revealing that the increase of lnOBS by one unit led to a greater reduction in SUA levels in men compared to women. We analyzed and speculated that several factors may contribute to this phenomenon. Firstly, relevant literature indicates that men have higher levels of physical activity compared to women. This higher level of physical activity helps men manage their weight more effectively, resulting in lower obesity rates among men compared to women. Additionally, in the OBS index, physical activity acts as an antioxidant, while obesity is a pro-oxidant. This may explain why uric acid levels decrease more rapidly in men than in women (64, 65). Moreover, sex-specific differences in physiological responses of the respiratory, musculoskeletal, and cardiovascular systems to physical activity result in a higher prevalence of metabolic syndrome in women (66). Metabolic syndrome, characterized by insulin resistance, enhances uric acid reabsorption and reduces uric acid excretion through upregulation of urate transporter 1 (URAT1) expression (67). Secondly, sex hormones also influence blood uric acid levels (68). Studies have shown that testosterone can directly regulate uric acid excretion by affecting the function of the URAT (69). Additionally, the binding degree of testosterone to hormone-binding globulin can also impact blood uric acid levels (70). Estradiol can promote intestinal urate excretion by regulating the intestinal ATP binding cassette subfamily G member 2 (ABCG2), thereby affecting uric acid levels (71). However, no significant difference was observed in the reduction of hyperuricemia prevalence among subgroups with different gender, ages, weight, diabetes statuses, hypertension status, and hyperlipidemia status. This is because the occurrence of hyperuricemia is the result of multiple factors and may not be solely influenced by oxidative balance. Genetic background (72), physiological factors (73), metabolic factors (74), medication effects (75), and environmental factors (76) may all play significant roles in the development of hyperuricemia. Therefore, although OBS can affect uric acid levels, relying solely on OBS regulation may not be sufficient to significantly reduce the incidence of hyperuricemia. This suggests that a comprehensive approach considering multiple factors is necessary for effective intervention and prevention.

This study possesses several strengths. Firstly, it is the first to establish associations between OBS and SUA levels as well as hyperuricemia within a US population. Secondly, the utilization of data from the NHANES, a nationwide population-based sample acquired through standardized protocols, enhances the robustness of our findings. The stratified, multistage sampling method employed in the NHANES ensures the representation of the noninstitutionalized population, thus increasing the external validity of our results. Thirdly, sophisticated statistical methods were employed to ensure comprehensive and reliable outcomes. Complex models were developed, accounting for multiple confounders, and OBS were adjusted to accommodate continuous and categorical variables, mitigating potential effects on the observed associations. Additionally, subgroup analyses were conducted to explore the potential influence of other factors on the association between OBS and SUA levels as well as hyperuricemia. However, our study also has limitations. Firstly, due to its cross-sectional design, we cannot infer a causal relationship between OBS and SUA levels as well as hyperuricemia. Prospective studies are needed to further validate the predictive value of OBS in hyperuricemia. What’s more, the self-reported dietary and lifestyle data in the NHANES database have limitations, including recall bias and social desirability bias. These biases may affect the accuracy of the data and the reliability of the study results, so they should be considered when interpreting the findings. Lastly, while our study revealed a significant difference in uric acid reduction between genders, no significant difference was observed in the reduction of hyperuricemia prevalence. However, previous relevant trials have suggested a sex difference. Therefore, further studies are warranted to elucidate this correlation.

## Conclusion

In conclusion, findings from a nationally representative sample of US adults indicated a negative correlation between OBS and SUA levels as well as hyperuricemia. Both an antioxidant-rich diet and improvements in lifestyle were found to be crucial in reducing SUA levels and decreasing the prevalence of hyperuricemia. Furthermore, we observed that the negative correlation between OBS and SUA levels was more pronounced among male participants compared to female participants. Future research should explore the mechanisms behind gender differences in the relationship between OBS and uric acid levels, particularly in the metabolism of antioxidants and pro-oxidants. Investigating how gender differences influence the occurrence and development of hyperuricemia is also essential. More randomized controlled trials are needed to assess whether improving OBS through dietary changes, such as increasing antioxidant intake or reducing pro-oxidant exposure, can effectively lower uric acid levels and reduce hyperuricemia incidence. Additionally, long-term cohort studies are necessary to determine the lasting impact of OBS on uric acid levels and the development of hyperuricemia over time, as well as to evaluate potential cumulative effects.

## Data availability statement

Publicly available datasets were analyzed in this study. This data can be found here: https://www.cdc.gov/nchs/nhanes/.

## Ethics statement

The studies involving humans were approved by National Center for Health Statistics Ethics Review Board. The studies were conducted in accordance with the local legislation and institutional requirements. The participants provided their written informed consent to participate in this study.

## Author contributions

YY: Writing – original draft, Writing – review & editing. ZW: Writing – original draft, Writing – review & editing. ZA: Formal analysis, Methodology, Project administration, Validation, Writing – review & editing. SL: Conceptualization, Funding acquisition, Investigation, Methodology, Project administration, Validation, Writing – review & editing.
